# β-Carotene prevents weaning-induced intestinal inflammation by modulating gut microbiota in piglets

**DOI:** 10.5713/ajas.19.0499

**Published:** 2019-12-24

**Authors:** Ruonan Li, Lingqian Li, Pan Hong, Wuying Lang, Junnan Hui, Yu Yang, Xin Zheng

**Affiliations:** 1 College of Animal Science and Technology, Jilin Agricultural University, Changchun 2888, China; 2 Key Laboratory of Functional Protein Research of Guangdong Higher Education Institutes, Institute of Life and Health Engineering, College of Life Science and Technology, Jinan University, Guangzhou 510632, China

**Keywords:** β-Carotene, *Prevotella*, Weaning, Inflammation, Gut Microbiota

## Abstract

**Objective:**

Weaning is an important stage in the life of young mammals, which is associated with intestinal inflammation, gut microbiota disorders, and even death. β-Carotene displays anti-inflammatory and antioxidant activities, which can prevent the development of inflammatory diseases. However, whether β-carotene can affect intestinal microbiota remains unclear.

**Methods:**

Twenty-four piglets were distributed into four groups: the normal suckling group (Con), the weaning group (WG), the weaning+β-carotene (40 mg/kg) group (LCBC), and the weaning+β-carotene (80 mg/kg) group (HCBC). The serum, jejunum, colon, and faeces were collected separately from each group. The effects of β-carotene on the phenotype, overall structure, and composition of gut microbiota were assessed in weaning piglets.

**Results:**

The results showed that β-carotene improved the growth performance, intestinal morphology and relieved inflammation. Furthermore, β-carotene significantly decreased the species from phyla *Bacteroidetes* and the genus *Prevotella*, and *Blautia*, and increased the species from the phyla *Firmicutes* and the genera *p-75-a5*, and *Parabacteroides* compared to the WG group. Spearman’s correlation analysis showed that *Prevotella* and *Blautia* were positively correlated, and *Parabacteroides* and *Synergistes* were negatively correlated with the levels of interleukin-1β (IL-1β), IL-6, and tumour necrosis factor-α (TNF-α), while *p-75-a5* showed negative correlation with IL-6 in serum samples from piglets.

**Conclusion:**

These findings indicate that β-carotene could alleviate weaning-induced intestinal inflammation by modulating gut microbiota in piglets. *Prevotella* may be a potential target of β-carotene in alleviating the weaning-induced intestinal inflammation in piglets.

## INTRODUCTION

Weaning is a critical and complex event in a pig’s life due to abrupt changes of physiological, dietary and environmental conditions, and because of its undeveloped intestinal and immune systems. It is generally associated with systemic stress responses, including transient anorexia, intestinal infections, gut microbiota disorders and even death [1]. As a result, weaning stress causes huge economic loss to the swine industry. The processes of digestion, absorption, and immunity are closely linked to the intestines, and the intestinal tissue plays important roles in maintaining homeostasis and normal functioning in animal health, growth and development [2].

The gut microbiota of mammals, such as pigs, has numerous roles benefiting the host, including maintenance of physiological functions of the intestinal villi, regulation of immune responses, protection from pathogenic bacteria, digestion and fermentation of carbohydrates and production of vitamins [3]. Furthermore, a recent report demonstrated that weaning induced a shift in the intestinal microbiome due to abrupt dietary shifts [4]. During the suckling period, milk provides an environment for the population of *Lactobacillus*. At this time, *Bacteroides*, *Bifidobacterium*, *Lactobacillus*, and *Clostridium* are all established. However, piglets suffer from significant imbalances of intestinal microbiota, including decreased populations of *Lactobacillus* and *Ruminococcaceae*, increased facultative anaerobes such as *Escherichia coli* and *Clostridium* spp., and loss of microbial diversity on weaning [5].

β-Carotene is a natural carotenoid, present in many fruits and vegetables. It acts as a precursor to vitamin A, by forming two molecules of vitamin A in the enterocytes. β-Carotene has antioxidant, anti-cancer and anti-cardiovascular-disease activities [6]. Over the past few decades, β-carotene is shown to prevent the development of inflammatory diseases and enhances many facets of the immune system [7]. It can also enhance mucosal immunoglobulin A induction in the small intestines of weanling mice [8]. In addition, β-carotene can inhibit the expression pro-inflammatory genes by suppressing nuclear factor κB (NF-κB) activation in lipopolysaccharide-stimulated macrophages [9]. Our previous study had found that β-carotene has the ability to attenuate weaning-induced oxidative injury and apoptosis in the jejunum of piglets [6]. Lee et al [10] reported that vitamin A may combat murine norovirus infection by modulating gut microbiota. Nevertheless, reports concerning the effect of β-carotene on gut inflammation and microbiota in piglets under weaning-stress are limited.

In the present study, we investigated whether β-carotene can protect against weaning-induced intestinal inflammation and we also determined the influence of bacteria during the β-carotene intervention. The results showed that β-carotene attenuates weaning-induced intestinal inflammation by altering the intestinal microbiota of piglets. Our study provides a new insight into the anti-inflammatory mechanism of β-carotene.

## MATERIALS AND METHODS

### Animals and treatments

All experiments involving animals were conducted in accordance with the guidelines of the animal ethical committee of Jilin University (No. 201705001). Male Jun Mu No. 1 white piglets (Sanjiang White Pig×Seghers hybrid, n = 24) were selected from 4 litters and randomly divided into four groups (n = 6/group): the control group (Con, normal suckling), the weaning group (WG, piglets weaned on d 21), the weaning+ β-carotene (40 mg/kg body weight) group (LCBC, oral supplementation of β-carotene from d 12 to d 26, weaned on d 21), and the weaning+β-carotene (80 mg/kg body weight) group (HCBC, oral supplementation β-carotene from d 12 to d 26, weaned on d 21). A basal diet was formulated according to National Research Council (2012) standards for the nutrient requirements of swine without antibiotics (Table 1).

Piglets were weighed at the beginning and the end of the experiment individually, and the average daily gain (ADG) were calculated. Faecal consistency within each group was visually assessed at 8:00 each day according to the method described by Hill et al [11] from d 12 to d 26. The scoring system for consistency was as follows: 1 = very firm, 2 = medium firm, 3 = moderately loose, 4 = very loose, and 5 = thin and watery. When the faeces were thin and watery, the occurrence of diarrhoea was scored. The blood was collected from the jugular vein, and serum was separated by centrifugation at 3,500 rpm for 15 min on d 5 after weaning. The faeces from each group (n = 6/group) were collected into 2 mL sterile tubes on d 26 to assess the gut microbiota (randomly selected 3 samples from the 6 collected/group). The piglets were then slaughtered using electrical stunning, and the intestinal segments (the middle of the jejunum and colon) were collected and instantly fixed in 10% paraformaldehyde for histology, and the rest of the tissues were frozen in liquid nitrogen and stored at −80°C.

### Intestinal morphology

Intestinal morphology was analysed using haematoxylin and eosin (H&E) staining. In brief, piglet jejunum and colon were fixed with 4% paraformaldehyde, dehydrated and embedded in paraffin blocks, before sectioning and staining with H&E. The sections were examined under a PreciPoint M8 microscope (M8, PreciPoint, Freising, Germany). Villus height was measured in at least 10 villi from each pig and averaged. Data were analysed using digital microscope M8 and photographed at 200× magnification.

### Enzyme-linked immunosorbent assay

Blood was collected from the jugular vein and serum was collected by centrifugation (3,500 rpm, 15 min) and the levels of cytokines, like tumour necrosis factor-α (TNF-α), interleukin-1β (IL-1β) and IL-6 were estimated using enzyme-linked immunosorbent assay (ELISA) kits (R&D systems, Minneapolis, MN, USA). All assays were conducted according to the manufacturer’s instructions.

### Real-time–polymerase chain reaction

The expression of TNF-α, IL-1β, and IL-6 mRNA in the jejunum and colon were quantitated by real-time (RT)–polymerase chain reaction (PCR) as described by Ren et al [12]. In brief, total RNA was extracted using Trizol reagent (Invitrogen, Carlsbad, CA, USA) according to the manufacturer’s instructions, and the yield and purity of the extracted RNA were determined using a NanoDrop 2000 spectrophotometer (Thermo Fisher Scientific, Waltham, MA, USA). cDNA was synthesised using the PrimeScript RT reagent kit (Takara, Dalian, China), and subjected to RT-PCR with SYBR Premix Ex Taq II (Tli RNaseH Plus, Takara, China) using the StepOne PlusTM RT-PCR system (Applied Biosystems, Foster City, CA, USA). The relative mRNA expression levels were calculated using the 2–ΔΔCt relative quantification method. The primer sequences used are as follows:

TNF-α: F: CATCGCCGTCTCCTACCA, R: CCCAGATT CAGCAAAGTCCA;
IL-1β: F: TGAAGTGCCGCACCCAAAACCT, R: CGGC TCCTCCTTTGCCACAATCA;
IL-6: F: ATGAACTCCCTCTCCACAAGC, R: TGGCTT TGTCTGGATTCTTTC;
β-actin: F: CTGCGGCATCCACGAAACT, R: ATGAAGT GCTGGGACACC

### Protein extraction and western blotting

Protein extraction was carried out following our previously reported methods [6]. Total protein was loaded onto 4% to 10% gradient gels for sodium dodecyl sulfate-polyacrylamide gel electrophoresis, and the separated protein bands were transferred to poly vinylidene fluoride membranes (Millipore, Bedford, MA, USA). The membranes were blocked by incubating in 5% skimmed milk (Pierce, Rockford, IL, USA), washed three times for 10 min each, in Tris-buffered saline containing 0.1% Tween-20. After this, the membranes were incubated with anti-NF-kB p65, anti-phospho-NF-kB p65 (phosphor S536) (Abcam, Cambridge, UK), anti-IκBα, anti-phosphor-IκBα (phosphor Ser32) (Cell Signaling Technology, Danvers, MA, USA) antibodies separately, overnight. The next day, membranes were incubated with horseradish peroxidase-conjugated secondary antibody (Sigma-Aldrich, St. Louis, MO, USA). Immunostained proteins on the membranes were measured using the electrochemiluminescence (Pierce, USA) detection system. Densitometric analyses of the visible protein bands were done using Quantity One software (developed by BioRad Technical Service Department, Hercules, CA, USA; LSG.TechServ. US@BioRad.com).

### DNA extraction

A total of 250 mg of DNA was extracted from faecal samples from pigs on d 26 (n = 3/group, selected randomly from 6/group) using Fast DNA SPIN extraction kits (MP Biomedicals, Santa Ana, CA, USA) according to the manufacturer’s instructions. The DNA yield was measured using a NanoDrop ND-1000 spectrophotometer (Thermo Fisher Scientific, USA). Quality of the isolated DNA was confirmed by electrophoresing on 0.8% agarose gel. DNA was stored at −20°C for further analysis.

### Amplification and sequencing of 16S rRNA genes

The V3–V4 hypervariable regions of 16S rRNA gene were amplified by PCR using the barcoded fusion primers: 338F (5′-ACTCCTACGGGAGGCAGCA-3′) and 806R (5′-GG ACTACHVGGGTWTCTAAT-3′). For this, a 25 μL reaction containing 5 μL of Q5 High-Fidelity DNA Polymerase (New England Biolabs (Beijing) Ltd., Beijing, China) was set up. PCR amplicons were purified with Agencourt AMPure Beads (Beckman Coulter, Indianapolis, IN, USA) and quantified using the PicoGreen dsDNA Assay Kit (Invitrogen, USA). The amplicons were pooled and normalized, and then paired-end 2×300 bp sequencing was performed using the Illumina MiSeq platform with the MiSeq Reagent Kit v3 at Shanghai Personal Biotechnology Co., Ltd. (Shanghai, China).

Sequencing data for the 16S rRNA was deposited in the SRA database under GenBank accession NO. SRP155827.

### Bioinformatics and statistical analysis

Sequence data analyses were conducted using quantitative insights into microbial ecology (QIIME, v1.8.0, University of Colorado, Denver, CO, USA) according to Caporaso et al [13]. The low-quality sequences were excluded with the following criteria: lengths of <150 bp and average Phred scores of <20, and containing ambiguous bases and mononucleotide repeats of >8 bp. Paired-end reads were assembled using FLASH. The remaining high-quality sequences were clustered into operational taxonomic units (OTUs) at 97% sequence identity by UCLUST (Edgar [14]). OTU taxonomic classification was conducted by BLAST and the OTUs containing more than 99.999% of total sequences across all samples were reserved.

All results are presented as mean values±standard deviation. Data were analysed with SPSS 19.0 using one-way analysis of variance (ANOVA). Differences among groups were evaluated for significance with the comparable variances, followed by least significant difference and Tukey’s tests. GraphPad Prism 7.0 software (GraphPad Software, La Jolla, CA, USA) was used for constructing graphs. Statistical significance is defined when p values are less than 0.05.

QIIME (v1.8.0, USA) and R packages (v3.2.0, Bell Labs Technology Showcase, Murray Hill, NJ, USA) were mainly used for sequence data analysis. The alpha diversity indices, including abundance-based coverage estimator (ACE) and Chao1 richness estimators, and the Shannon diversity index were calculated using the OTU table in QIIME. Venn diagram were used to compare OTUs between groups. To further study the mechanism of β-carotene supplementation in reducing gut inflammation, the intestinal bacterial abundance and diversity in faeces were determined. Bacterial abundance was shown by Chao1 and ACE [15]. Shannon analysis provided a comprehensive picture of the richness and evenness of the bacterial community in a sample [16]. The bacterial diversity was indicated by the number of OTUs. Beta diversity was calculated using weighted UniFrac distance and was displayed by principal component analysis (PCoA). The significance of microbiota structure differentiation among groups was assessed by PERMANOVA (permutational multivariate analysis of variance) and ANOSIM (analysis of similarities) using the R package “vegan”. Taxa abundances at different taxonomies were statistically compared among groups by Metastats and visualized as a violin diagram combined with a box diagram.

## RESULTS

### Effects of β-carotene on growth performance, intestinal morphology, and the levels of inflammatory cytokines in serum

**Figure 1:**
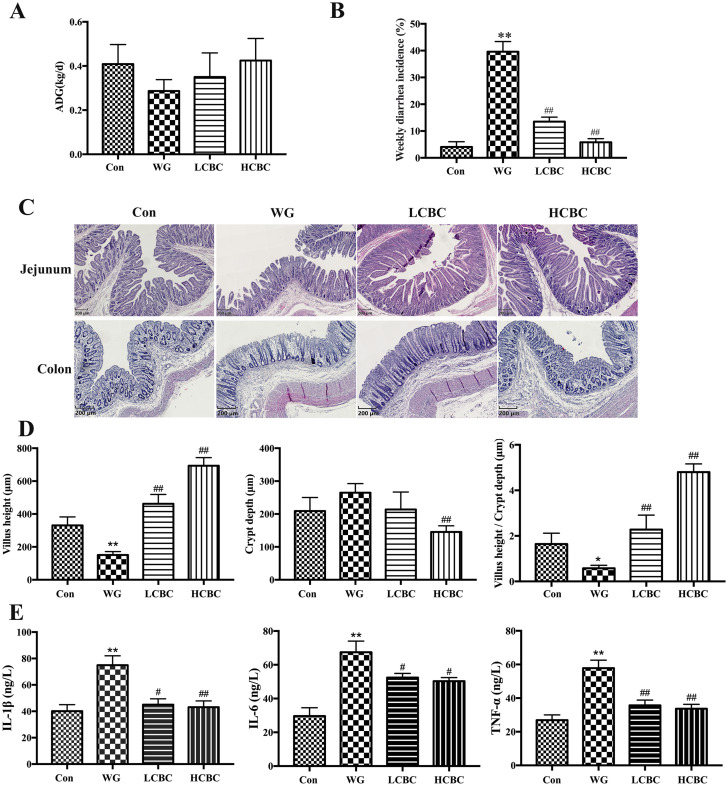
(A) Piglets were weighed daily, from d 12 to d 26 after birth, and average daily gain (ADG) was calculated; n = 6/group. (B) Faecal consistency within each group was visually assessed at 8:00 each day from d 12 to d 26 after birth, n = 6/group. The weekly diarrhoeal incidence was estimated. (C, D) The jejunum and colon in different groups were collected, fixed in 4% paraformaldehyde and embedded in paraffin wax. Sections of 5 μm thickness were cut and intestinal morphology was studied after staining with hematoxylin and eosin (H&E). (E) Blood samples were collected from the jugular vein and the serum concentrations of inflammatory cytokines TNF-α, IL-1β, and IL-6 were measured by ELISA; n = 6/group. TNF-α, like tumour necrosis factor-α; IL-1β, interleukin-1β; IL-6, interleukin-6; ELISA, enzyme-linked immunosorbent assay; Con, piglets with normal suckling; WG, piglets weaned on d 21; LCBC, piglets with supplementation of β-carotene (40 mg/kg) from d 12 to d 26, weaned on d 21; HCBC, piglets with supplementation of β-carotene (80 mg/kg) from d 12 to d 26, weaned on d 21. The results are expressed as the mean±standard deviation of three separate experiments. * Represents p<0.05, ** represents p<0.01 compared to Con group, and # represents p<0.05, ## represents p<0.01 compared to WG group.

In this work, we measured the ADG and jejunum morphology in piglets. As shown in Figure 1A, the ADG in the WG group was lower than that in the Con group; treatment with β-carotene slightly increased the ADG, but the difference was not significant. The WG group piglets showed a higher weekly diarrhoeal incidence compared to Con group, and this was significantly alleviated by the treatment with β-carotene (p< 0.01) (Figure 1B). Jejunum villus height (p<0.01) and the ratio of villus height to crypt depth (p<0.05) of the WG group piglets were significantly lower than those in the piglets from the Con group, and they were much higher (p<0.01) in the HCBC group (Figure 1C, D). Compared to the Con group, the architecture of the colonic crypts in the WG group was disrupted, and showed distorted, dilated, decreased structures, and in some cases, crypts were absent. Such disruptions in the HCBC and LCBC groups were less severe compared to that in the WG group (Figure 1C).

When the levels of inflammatory cytokines in the serum of piglets were estimated (Figure 1E), WG group showed significantly (p<0.01) higher levels of TNF-α, IL-1β, and IL-6 compared to those in the Con group, while β-carotene markedly reduced the levels of these cytokines. This result suggests that β-carotene has the ability to reduce the inflammatory factors (TNF-α, IL-1β, and IL-6) in serum.

### Dietary β-carotene improved the relative expression of mRNA of inflammatory cytokines

**Figure 2:**
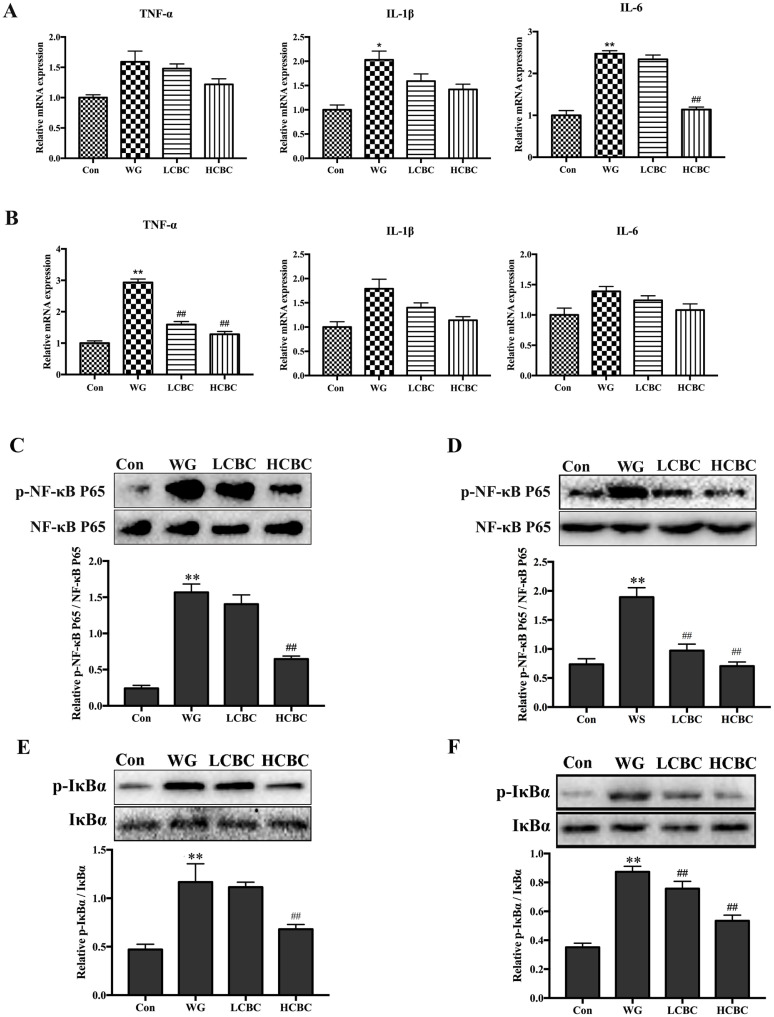
The levels of TNF-α, IL-1β, and IL-6 mRNA in jejunum (A) and colon (B) were quantified with RT-PCR. The expression of phosphorylated and total NF-κB p65 protein in jejunum (C) and colon (D), the expression of phosphorylated and total IκBα protein in jejunum (E) and colon (F) were detected by western blots. TNF-α, like tumour necrosis factor-α; IL-1β, interleukin-1β; IL-6, interleukin-6; RT-PCR, real-time polymerase chain reaction; NF-κB, nuclear factor κB; IκBα, inhibitor of NF-κB alpha. Con, the piglets with normal suckling; WG, piglets weaned on d 21; LCBC, piglets with supplementation of β-carotene (40 mg/kg) from d 12 to d 26, weaned on d 21; HCBC, piglets with supplementation of β-carotene (80 mg/kg) from d 12 to d 26, weaned on d 21. The results are expressed as the mean±standard deviation. of three separate experiments. * Represents p<0.05, ** represents p<0.01 compared to Con group, and # represents p<0.05, ## represents p<0.01 compared to WG group.

To find out whether β-carotene can reverse the development of weaning-induced intestinal inflammatory responses, the expression levels of inflammatory genes (TNF-α, IL-1β, and IL-6) in the jejunum and colon were measured (Figure 2A, B). The results showed that the mRNA levels of IL-1β and IL-6 increased significantly (p<0.05, p<0.01) in the jejunum of the animals from the WG groups compared to those from the Con group, while the TNF-α mRNA increased (p<0.01) in the colon of the WG group. β-Carotene supplementation reversed the changes in the mRNA levels of IL-6 in the jejunum and TNF-α in the colon; however, it had no significant effects on the expression of other inflammatory genes, like TNF-α, IL-1β in the jejunum and IL-1β and IL-6 in the colon.

### β-Carotene suppressed weaning-induced NF-κB pathway activity in jejunum and colon tissues

We analysed the effects of β-carotene on the NF-κB pathway in the jejunum and colon on western blots (Figure 2C–F). The results indicated that the IκB and NF-κB p65 were activated significantly (p<0.01) in both jejunum and colon, upon weaning. β-Carotene, at the dose of 80 mg/kg body weight, brought about substantial attenuation of IκBα phosphorylation and the translocation of p65 (p<0.01). These results suggested that β-carotene suppressed the phosphorylation of IκBα and NF-κB p65 proteins in the weanlings.

### β-Carotene alters the profiles of faecal microbiota in weaning piglets

**Figure 3:**
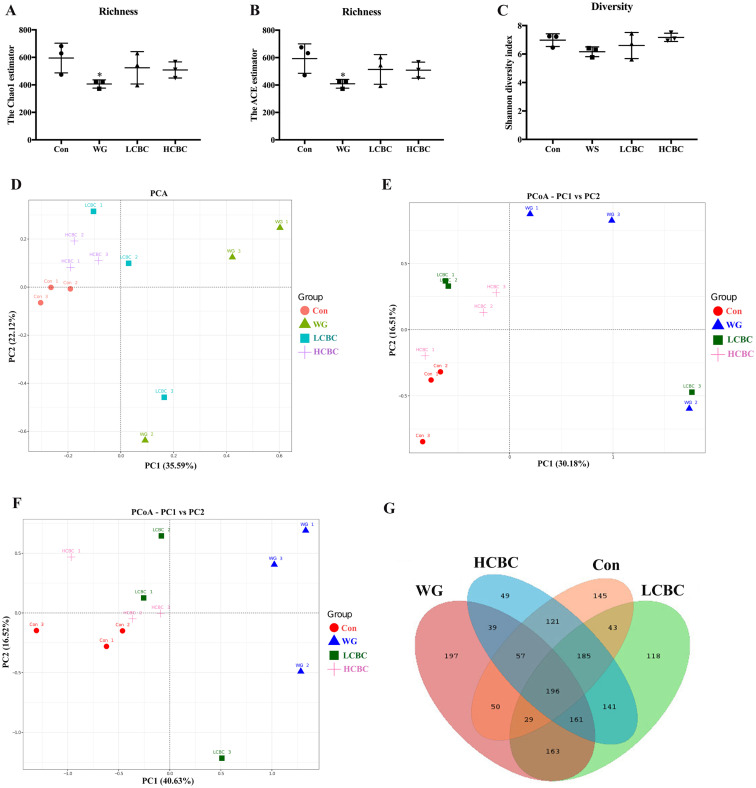
β-Carotene treatment improves the profiles of faecal microbiota in newly weaned piglets. Bacterial genomic DNA was extracted from the faeces collected on day 26 from each group, and 16S rRNA sequence analysed. The Chao1 estimator (A), ACE estimator (B), and the Shannon diversity index (C) were evaluated through alpha diversity analysis. Principal component analysis (PCA), and beta-diversity analysis (D), principal component analysis (PCoA) score plot based on unweighted UniFrac (E) and weighted UniFrac (F) were conducted to explore the similarities of faecal microbiota community structure between different samples. (G) Venn diagram showing the unique and shared operational taxonomic units (OTUs) in the faecal bacteria among four different treatments. Con, piglets with normal suckling; WG, piglets weaned on d 21; LCBC, piglets with supplementation of β-carotene (40 mg/kg) from d 12 to d 26, weaned on d 21; HCBC, piglets with supplementation of β-carotene (80 mg/kg) from d 12 to d 26, weaned on d 21. * Represents p<0.05 compared to Con group.

High-throughput 16S rRNA gene sequencing produced a total of 429,198 good-quality sequences from 12 samples. Rarefaction curves and rank abundance curves have shown that the most faecal microbiota in samples were captured based on the current sequencing depth and the data can be used for further analysis (Supplementary Figure S1). Next, we analysed the community of faecal microbiota in piglets affected by weaning-induced intestinal inflammation with and without β-carotene intervention. Alpha-diversity analysis indicated that the values of Chao1, ACE were significantly decreased (p<0.05) in the WG group compared to the Con group. After treatment with 80 mg/kg β-carotene, these values improved relative to the WG group. However, the values of Shannon index were not significantly affected by weaning or β-carotene supplementation (Figure 3A–C).

Furthermore, as shown in Figure 3D–F, certain analyses were conducted to investigate the similarities in the structure of faecal microbiota among different samples. The PCoA plot indicated that the structure of faecal microbiota in the WG group was different from that in the Con group along the PC1 axis, and a structural shift was also seen in most of the β-carotene-supplemented piglets compared to the WG group. In addition, the results of the unweighted pair group method with arithmetic mean based on weighted and unweighted Unifrac also showed overt changes in the composition of faecal microbiota in the LCBC and HCBC groups, compared to the WG group (Supplementary Figure S2), and this is consistent with the PCoA results. Together, these results indicated that weaning caused distinct differences in the distribution of the bacterial community of the piglets, and β-carotene supplementation reduced these differences. Venn diagrams were constructed to represent the shared richness (196 OTUs) among the Con, WG, LCBC, HCBC groups (Figure 3G). Lot more unique OTUs were detected in the faecal samples from all these groups as well. These results showed that weaning decreased the diversity of faecal microbiota in piglets and the microbial community structure also changed with weaning. β-Carotene supplementation effectively improved the abundance and diversity of the intestinal bacteria in weaning piglets.

### Effect of dietary β-carotene on faecal microbiota composition in weaning piglets

**Figure 4:**
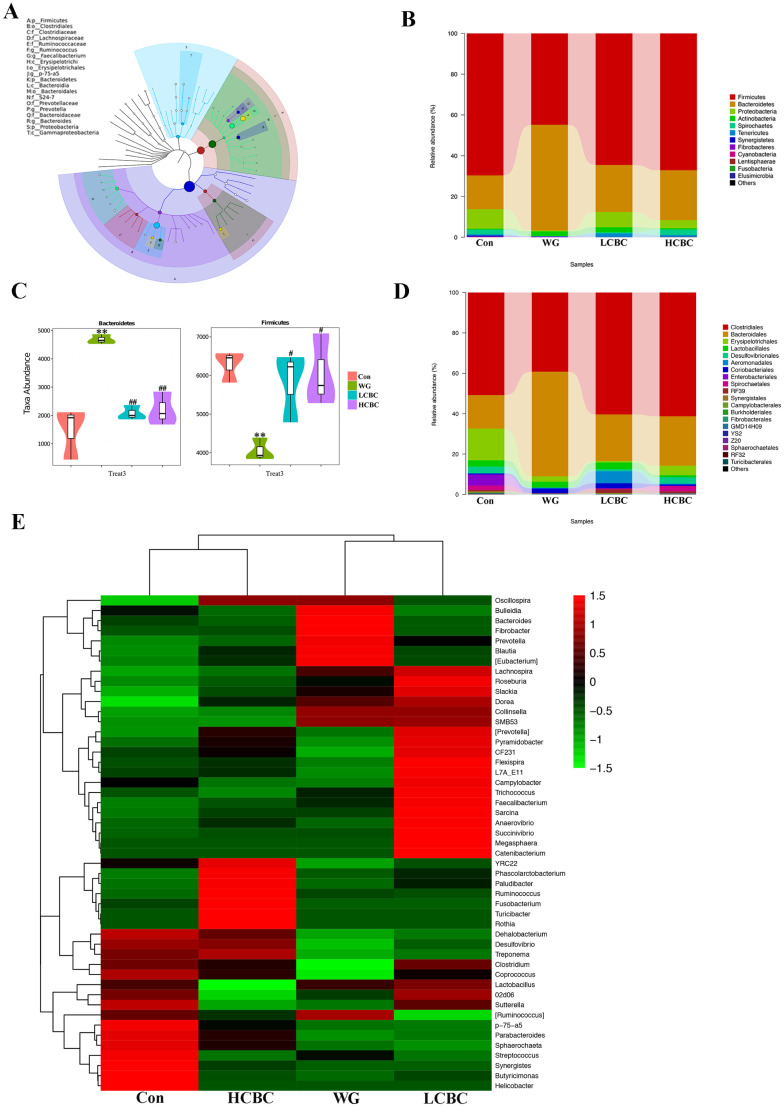
(A) The overall classification based on GraphlAn was used to visualize the taxonomy and abundances of the intestinal microbiota among the top 20 most abundant groups, as inferred by GraphlAn. (B) The composition and abundance distribution of each group at the phylum level were shown using quantitative insights into microbial ecology (QIIME) software. (C) Pair-wise comparisons at the phylum level conducted to determine the sequences shared between two groups, and presented as pair-wise comparisons in the form of a violin diagram combined with a box diagram, by Metastats analysis. (D) The composition and abundance distributions at the orders level for each group, shown using QIIME software. (E) Heatmap of the abundance of bacteria at the genus level for different groups. The top 50 most-abundant genera were clustered and the heat map was drawn, using R software. The red colour represents the genera with higher abundance, and the green colour represents those with lower abundance. Con, piglets with normal suckling; WG, piglets weaned on d 21; LCBC, piglets supplemented with β-carotene (40 mg/kg) from d 12 to d 26, weaned on d 21; HCBC, piglets supplemented with β-carotene (80 mg/kg) from d 12 to d 26, weaned on d 21. * represents p<0.05, ** represents p<0.01 compared to Con group, and # represents p<0.05, ## represents p<0.01 compared to WG group.

**Figure 5:**
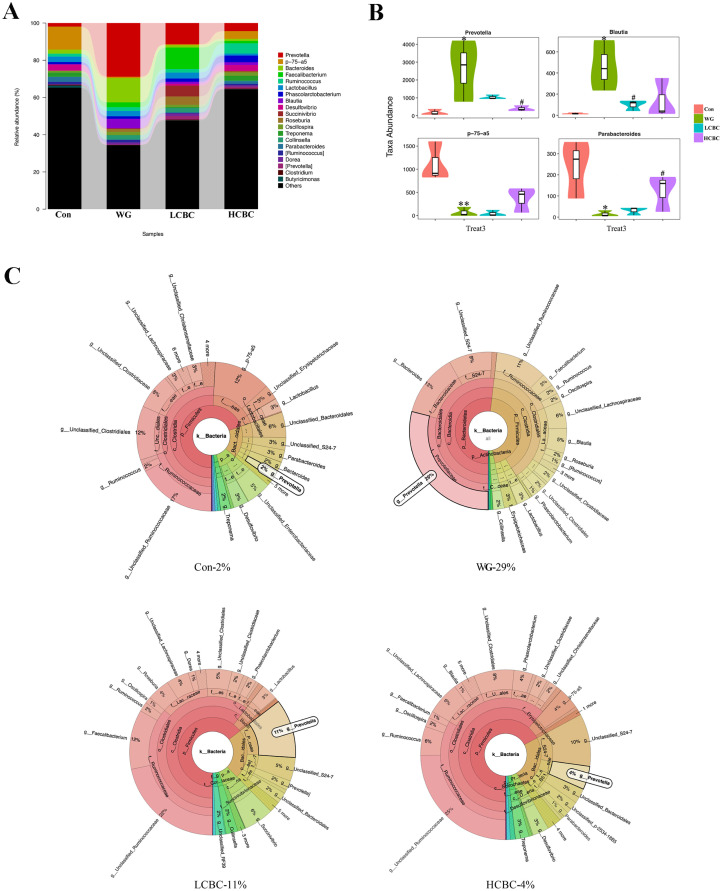
(A) The composition and abundance distributions of each group of gut microbiota at the genus level shown using quantitative insights into microbial ecology (QIIME) software. (B) Pair-wise comparisons at the genus level to determine the sequence shared between two groups, and presented as pair-wise comparisons in the form of a violin diagram combined with a box diagram, by Metastats analysis. (C) β-Carotene prevents weaning-induced Prevotella enrichment. The taxonomic composition of bacterial community among different groups analysed using Krona software. Con, piglets with normal suckling; WG, piglets weaned on d 21; LCBC, piglets with supplementation of β-carotene (40 mg/kg) from d 12 to d 26, weaned on d 21; HCBC, piglets with supplementation of β-carotene (80 mg/kg) from d 12 to d 26, weaned on d 21. * Represents p<0.05, ** represents p<0.01 compared to Con group, and # represents p<0.05, ## represents p<0.01 compared to WG group.

We detected 12 bacterial phyla and 76 genera of microflora among the piglets. The structure and composition of faecal microbiota were significantly altered by weaning. The results of the top 20 most abundant OTUs at all taxonomic levels in samples, as inferred by GraPhlAn, showed that *Firmicutes*, *Bacteroidetes*, *Proteobacteria* were the most abundant phyla, and *Ruminococcus*, *Faecalibacterium*, *p-75-a5*, *Prevotella*, and *Bacteroides* were the most abundant genera among the OTUs (Figure 4A). The taxonomic profiles indicated that the proportions of *Bacteroidetes* increased, and the abundance of *Firmicutes* decreased drastically in the WG group, while β-carotene significantly reversed these tendencies (Figure 4B, C). At the order level, weaning decreased the relative abundance of *Clostridiales* and increased those of *Bacteroidales*. Treatment with β-carotene restored the normal abundance of these orders (Figure 4D). At the genus level, bacterial taxa displayed obvious changes in the heat maps (Figure 4E). Genera including *Bulleidia*, *Bacteroides*, *Fibrobacter*, *Prevotella*, *Blautia*, [*Eubacterium*] and *Clostridium* increased by weaning compared to suckling stages and partially reversed by β-carotene (40 mg/kg and 80 mg/kg) supplementation. Genera *Oscillospira*, *Lactobacillllus*, *02d06* and *Sutterella*, switched by weaning and partially reversed by β-carotene (40 mg/kg) supplementation. Similarly, the abundance of *Lachnospira*, *Dorea*, *Collinsella*, *Smb53*, *Dehalobacterium*, *Desulfovibrio*, *Treponema*, *p-75-a5*, *Parabacteroides*, and *Sphaerochaeta* were significantly switched by weaning and partially reversed by the treatment with β-carotene at higher dose (80 mg/kg). As shown in Figure 5A–C, weaning increased the relative abundance of *Prevotella* and *Blautia*, and decreased that of *p-75-a5* and *Parabacteroides*, compared with the WG group. β-Carotene treatment reversed these changes in the abundance. Similarly, the abundance of *Prevotella* increased sharply in the WG group (29%) but was almost restored to its normalcy in the HCBC group (4%), while in the Con group it was at the lowest (2%).

### Relationship between bacterial community and the levels of inflammatory cytokines in serum

**Figure 6:**
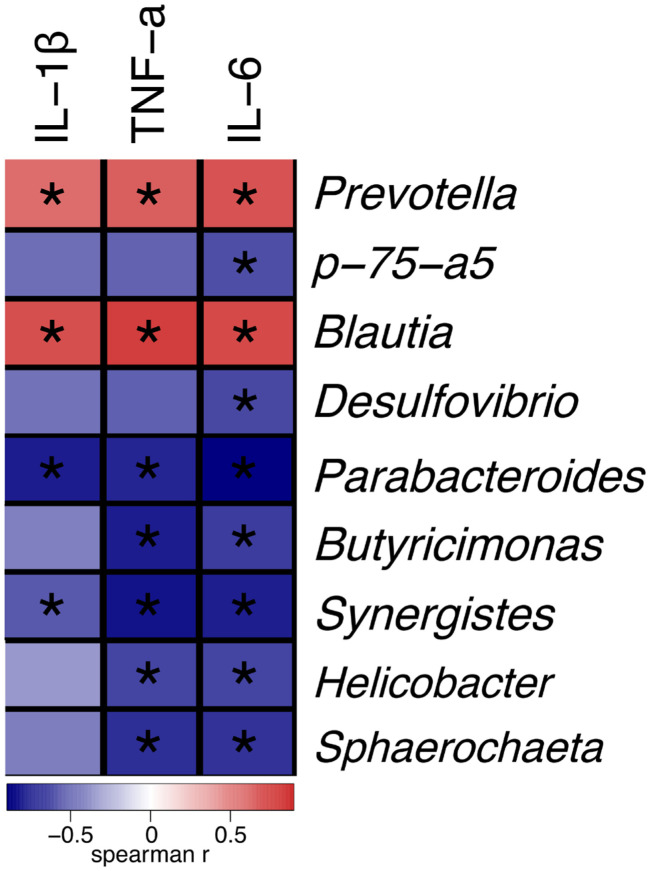
Spearman’s correlation between the faecal bacterial communities at the genus level and inflammatory cytokines in serum. The colours represent the correlation coefficient. Red represents a positive correlation, and blue, a negative correlation; a darker colour represents a stronger correlation, and a lighter colour indicates a weaker correlation. The square with “*” means p<0.05; smaller the p value, higher the credibility of the correlation.

Next, we analysed the relationship between bacterial community in the gut and the levels of inflammatory cytokines in the serum. Correlation analysis showed that the relative abundances of *Parabacteroides* and *Synergistes* were negatively correlated with IL-1β, IL-6, and TNF-α, while those of the *Prevotella* and *Blautia* showed positive correlations with IL-1β, IL-6, and TNF-α. In addition, *p-75-a5* showed negative correlations with IL-6 (Figure 6).

## DISCUSSION

Current information indicates that weaning could decline in concentrations of vitamin A levels in blood, and lowers the total antioxidant contents in the animal body. Subsequently, the animal may have reduced feed utilization efficiency, enhanced the risk for inflammation and diarrhoea, decreased immunity, disturbed dynamic balance of intestinal microbiota and other damages [17]. Several studies have reported that β-carotene could relieve inflammation [7,9], and supplementation with natural antioxidants, including tea polyphenols, vitamins E and C, and probiotics, could relieve stress and regulate the balance of intestinal microbiota [18]. Here, we demonstrate that β-carotene intervention can mitigate the weaning-induced intestinal inflammation, through restoration of the structure of gut microbiota, and decreased abundance of *Prevotella*.

In the present study, we found that weaning increased of the mRNA levels of inflammatory factors, including IL-1β, IL-6, and TNF-α in the intestinal tissues of piglets on d 5 of weaning, which were in accordance with previous studies [19]. β-Carotene intervention increased the ADG, alleviated weekly diarrhoeal incidence, protected the morphology and integrity of the intestinal tissue and suppressed the levels of proinflammatory cytokines (IL-1β, IL-6, and TNF-α) in serum. However, it is interesting that β-carotene supplementation had no significant effect on the expression levels of IL-1β and TNF-α mRNA in the jejunum, and IL-1β and IL-6 in the colon. Previous reports have shown that β-carotene can function not only at the transcriptional and post-transcriptional levels, but also at the translational and post-translational levels [20]. Dufour et al [21] reported that β-carotene can bind to β-lactoglobulin and influence protein modifications, indicating its ability to exert a regulatory role at the post-translational level. In this light, we speculate that β-carotene exerts its inhibitory effect on serum proinflammatory cytokines at the post-transcriptional level, rather than during transcription. Further experiments are needed to confirm this speculation, since these inhibitory effects of β-carotene on inflammation-related proteins may not be fully explained only at their mRNA levels.

Inhibitor of NF-κB kinase (IKK)/IκB/NF-κB signalling plays an important role in regulating inflammation. Activation of NF-κB signalling starts with the activation of the upstream IKK. Once activated, the NF-κB p65 subunit is released after IκB phosphorylation. Phosphorylated NF-κB p65 translocates into the nucleus to regulate target gene transcription, including numerous inflammatory mediators and cytokines, such as IL-1β, IL-6, and TNF-α [22]. Our previous study [23] found that β-carotene suppresses the production of IL-1β, IL-6, and TNF-α in lipopolysaccharide (LPS)-induced inflammation in macrophages. In the present study, we observed that the effect of β-carotene on the phosphorylation of IκB and NF-κB p65 was enhanced in the WG group, and treatment with β-carotene lowered the phospho-IκB and NF-κB p65 expression levels, and partly protected the intestinal tissue from weaning-induced inflammation. However, NF-κB is not the only transcription factor for the signaling and induction of pro-inflammatory cytokines. In addition to NF-κB, signal transducers and activators of transcription (STAT) and mammalian mitogen-activated protein kinase (MAPK) are also important transcription factors that are involved in immunity and inflammatory pathways [24]. In our previous study [23], we observed that β-carotene attenuated LPS-induced the release of pro-inflammatory cytokines and mediators through the suppression of the JAK2-STAT3 and JNK/p38 MAPK signal pathways in macrophages. In this work, we only investigated the NF-κB pathway of weaning-induced intestinal inflammation in the piglets. We also cannot fully exclude that the JAK2/STAT3 and MAPKs signalling pathways were inhibited by β-carotene or the negative regulators of JAK/STAT pathway were activated by β-carotene, such as suppressors of cytokine signaling family and protein tyrosine phosphatase, which inhibited JAK/STAT pathway.

To further explore whether β-carotene could prevent weaning-induced intestinal inflammation through modulating gut microbiota in piglets, we assessed the effects of β-carotene on faecal microbiota. The Chao1 index and the ACE index focus on the richness of the community. The Chao1 and ACE richness estimation indices determine the number of species actually present in the community [15]. Unlike the Chao1 and ACE indices, the Shannon index focuses on the community uniformity, and the Shannon diversity index considers the richness and uniformity of the community. The Shannon index is more sensitive to community richness and rare OTUs [16]. In our study, the faecal microbiota community results showed that the values of Chao1, ACE significantly decreased (p<0.05) in the WG group compared to the Con group, and β-carotene (80 mg/kg) improved them. However, the values of Shannon index were not significantly affected by weaning or β-carotene supplementation (Figure 3A–C). We speculate that it may because, in the weaned piglets, β-carotene has a greater influence on the community richness, rather than on uniformity of community of intestinal microflora.

*Ruminococcaceae* primarily inhabit the caecum and colon of animals, and produce anti-inflammatory metabolites, like short chain fatty acids. They are responsible for the degradation of diverse polysaccharides and fibres. Leclercq et al [25] reported a negative correlation between gut population of *Ruminococcaceae* and increased intestinal permeability. We observed the relative abundance of *Ruminococcaceae* and *Clostridiales* decreased in the weaned group but increased in response to β-carotene. This result was consistent with those reported by Scher et al [26]. In the present study, we found that β-carotene supplementation relieved the weaning-induced ADG decrease and high incidence of diarrhoea significantly (p<0.01), and reduced the weaning-induced intestinal disruption. It may be presumed that β-carotene improved growth performance and intestinal morphology of piglets by promoting *Ruminococcaceae* growth.

Lavy et al [27] demonstrated that β-carotene, as a prophylactic dietary supplement, reduced the effects of acid-induced enteritis in a rat model. In the current study, we identified that weaning significantly affected the microbiota composition and the relative abundance of intestinal microbiota. β-Carotene supplementation alleviated the gut microbiota imbalance resulting from weaning. Weaning also decreased the *Parabacteroides*, the beneficial intestinal bacteria associated with T-cell differentiation, by increasing and maintaining IL-10 [28], while β-carotene supplementation (80 mg/kg) markedly increased its relative abundance. Kverka et al [29] found that *Parabacteroides distasonis* attenuates colitis by modulating microbiota composition and immunity in mice. In fact, not only *P. distasonis*, but also *Parabacteroides* spp. were found to be drastically decreased in the inflamed tissues [30], similar to our results. We speculate that the anti-inflammatory role of β-carotene may be partly responsible for enhancing the relative abundance of *Parabacteroides*.

*Prevotella* was the most abundant genus in the post-weaning piglets. In our study, we showed that the relative abundance of *Prevotella* increased among the faecal flora of the WG group piglets, which was in agreement with the previous study by Pajarillo et al [31]. *Prevotella* is a beneficial microbe that is associated with a plant-rich diet. However, increasing evidence indicates that *Prevotella* is also associated with inflammatory conditions in the gut. Hofer [32] suggested that *Prevotella copri* has a role in the pathogenesis of rheumatoid arthritis, one of the most common autoimmune diseases. Animals colonized by *Prevotella copri* had greater distress than the controls in a mouse model of gut inflammation, consistent with a proinflammatory role of this organism [26]. Larsen [33] found that it stimulates the epithelial cells to produce IL-8, IL-6, and the chemokine ligand 20 (CCL20), which promotes mucosal T helper (Th17) immune responses and neutrophil recruitment. *Prevotella*-mediated mucosal inflammation leads to systemic diffusion of inflammatory mediators, bacteria and bacterial products, which, in turn may affect systemic disease outcomes. Our results found that β-carotene could limit the abundance of *Prevotella* to its normal level, indicating that *Prevotella* may have an important role in alleviating weaning-induced intestinal inflammation and benefit of its alleviation by β-carotene supplementation.

Thingholm et al [34] showed the higher abundance of *Blautia* in the faecal flora of patients with irritable bowel syndrome. In our study, weaning sharply increased the relative abundance of *Blautia* and decreased that of *p-75-a5*. These population dynamics were effectively restored to normalcy, by the β-carotene intervention. In summary, we found that weaning altered the microbiota composition and the relative abundance of intestinal microbiota, and treatment with β-carotene reduced the number of harmful bacteria and increased the beneficial bacteria in the intestine.

Significant correlations between certain gut bacteria and inflammatory cytokines in serum confirmed that pro-inflammatory cytokines in serum drastically altered the intestinal microbial composition. We found that *Parabacteroides* and *Synergistes* were negatively correlated with IL-1β, IL-6, and TNF-α, and *Blautia* and *Prevotella* were in positive correlation with these proinflammatory cytokines (Figure 6). These results suggested that β-carotene might exert its anti-inflammatory effects via cytokines in serum through shifting the balance in favour of beneficial bacterial communities. We speculate that improving the intestinal microbiota may be one of the anti-inflammatory strategy of β-carotene.

In conclusion, our study explored the protective effects of β-carotene on weaning-induced intestinal inflammation in piglets and its ability to regulate the gut microbiota. Overall structure and composition of the intestinal microbiota was altered by weaning, and it can be restored by β-carotene, particularly by limiting the relative abundance of *Prevotella*. Our results indicate that *Prevotella* may be a potential target for β-carotene intervention, for alleviation of weaning-induced intestinal inflammation. Our work provides a theoretical explanation for the well-recognized beneficial effects of dietary β-carotene in intestinal inflammation and distress, induced by weaning and other causes.

## Supplementary Information



## Figures and Tables

**Table 1:** Ingredient and chemical composition of the basal diet

Items	
Ingredients (g/kg)	
Maize	357
Extruded corn	210
Soybean meal	113
Extruded full-fat soybean	109
Fish meal	31
Spray-dried plasma protein	40.5
Dried whey	87
Soybean oil	19
Dicalcium phosphate	11
Limestone	5
Sodium chloride	1
L-lysine HCl	4.9
DL-methionine	1.6
Vitamin-mineral premix^1)^	10
Analysed composition (g/kg)	
Digestible energy^2)^ (MJ/kg, calculated)	14.4
Crude protein (measured)	223.57
Lysine (measured)	14.3
Methionine (measured)	3.6
Calcium (measured)	8.3
Total phosphorus (measured)	6.6

1) Provided per kilogram of diet: vitamin A, 8,000 IU; vitamin D, 2,000 IU; vitamin E, 40 IU; vitamin K_3_, 1.5 mg; vitamin B_1_, 1.5 mg; vitamin B_6_, 1.6 mg; biotin, 0.10 mg; niacin, 30 mg; pantothenic acid, 25 mg; Zn, 100 mg; Fe, 110 mg; Cu, 15 mg; Mn, 16 mg; I, 0.3 mg; Se, 0.3 mg.

2) Digestible energy was calculated from data provided by Feed Database in China (2012).
