# Supplementary material for: β-Carotene prevents weaning-induced intestinal inflammation by modulating gut microbiota in piglets
Source: Anim Biosci. 2019 Dec 24;34(7):1221–34. doi: 10.5713/ajas.19.0499 (PMC8255870; doi:10.5713/ajas.19.0499)
Supplement: Supplementary file 1 [file ajas-19-0499-suppl.pdf]

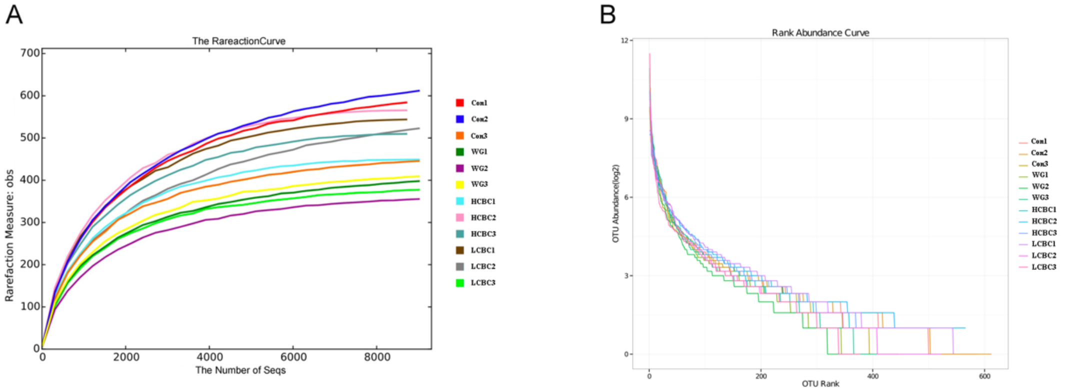

Figure S1. Rarefaction of sequencing data was evaluated by rarefaction (A) and rank abundance curve (B). It was observed that the rarefaction curves tended to be flat when the sequence number increased to 8000, rank abundance curves showed that the ideal richness and evenness of OTUs. These results indicating that the amount of sequencing data was reasonable.

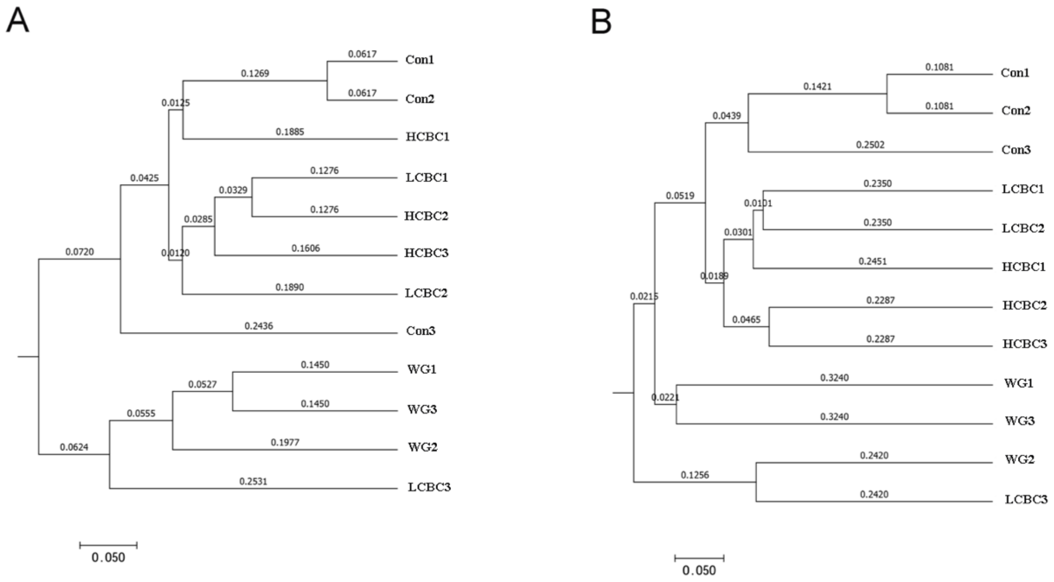

Figure S2. Cluster analysis using the unweighted pair group method with arithmetic mean (UPGMA) based on weighted Unifrac (A) and unweighted Unifrac (B) analysis.
